# Decreased gray matter volume in the right middle temporal gyrus associated with cognitive dysfunction in preeclampsia superimposed on chronic hypertension

**DOI:** 10.3389/fnins.2023.1138952

**Published:** 2023-05-12

**Authors:** Chaofan Sui, Hongwei Wen, Jingchao Han, Tao Chen, Yian Gao, Yuanyuan Wang, Linfeng Yang, Lingfei Guo

**Affiliations:** ^1^Department of Radiology, Shandong Provincial Hospital Affiliated to Shandong First Medical University, Jinan, Shandong, China; ^2^Key Laboratory of Cognition and Personality, Ministry of Education, Faculty of Psychology, Southwest University, Chongqing, China; ^3^Department of Medical Imaging, Jinan Stomatological Hospital, Jinan, Shandong, China; ^4^Department of Clinical Laboratory, Jinan Maternity and Child Care Hospital Affiliated to Shandong First Medical University, Jinan, Shandong, China; ^5^Department of Radiology, Binzhou Medical University, Yantai, Shandong, China; ^6^Department of Radiology, Jinan Maternity and Child Care Hospital Affiliated to Shandong First Medical University, Jinan, Shandong, China

**Keywords:** preeclampsia superimposed, chronic hypertension, voxel-based morphometry, gray matter volume, magnetic resonance imaging

## Abstract

**Introduction:**

The effects of preeclampsia superimposed on chronic hypertension (CHTN-PE) on the structure and function of the human brain are mostly unknown. The purpose of this study was to examine altered gray matter volume (GMV) and its correlation with cognitive function in pregnant healthy women, healthy non-pregnant individuals, and CHTN-PE patients.

**Methods:**

Twenty-five CHTN-PE patients, thirty-five pregnant healthy controls (PHC) and thirty-five non-pregnant healthy controls (NPHC) were included in this study and underwent cognitive assessment testing. A voxel-based morphometry (VBM) approach was applied to investigate variations in brain GMV among the three groups. Pearson’s correlations between mean GMV and the Stroop color-word test (SCWT) scores were calculated.

**Results:**

Compared with the NPHC group, the PHC and CHTN-PE groups showed significantly decreased GMV in a cluster of the right middle temporal gyrus (MTG), and the GMV decrease was more significant in the CHTN-PE group. There were significant differences in the Montreal Cognitive Assessment (MoCA) and Stroop word scores among the three groups. Notably, the mean GMV values in the right MTG cluster were not only significantly negatively correlated with Stroop word and Stroop color scores but also significantly distinguished CHTN-PE patients from the NPHC and PHC groups in receiver operating characteristic curve analysis.

**Discussion:**

Pregnancy may cause a decrease in local GMV in the right MTG, and the GMV decrease is more significant in CHTN-PE patients. The right MTG affects multiple cognitive functions, and combined with the SCWT scores, it may explain the decline in speech motor function and cognitive flexibility in CHTN-PE patients.

## 1. Introduction

Hypertensive disorder of pregnancy (HDP), including preeclampsia/eclampsia, gestational hypertension, chronic hypertension, and preeclampsia/eclampsia superimposed on chronic hypertension, continues to be among the major causes of maternal and fetal morbidity and mortality around the world (International Lobour Organization [ILO], 2013; Garovic et al., 2020). HDP affects the mother and fetus during the disease course and is linked to future morbidity and mortality of cardiovascular disease and stroke (Garrido-Gimenez et al., 2020). Chronic hypertension is a dangerous condition for pregnant women and becomes more common as women become pregnant in their later years (Maducolil et al., 2020). In the United States, approximately 26% of women with chronic hypertension have superimposed preeclampsia, a type of HDP (Bramham et al., 2014, 2020). Women who develop superimposed preeclampsia are at an increased risk for preterm delivery, placental abruption (Boriboonhirunsarn et al., 2017), acute renal failure, cardiovascular or coagulation dysfunction, stroke, or even maternal death (Abalos et al., 2014; Bramham et al., 2020). Investigating the pathogenesis of preeclampsia superimposed on chronic hypertension (CHTN-PE) is therefore crucial.

The effects of pregnancy on the structure and function of the human brain are poorly understood. The voxel-based morphometry (VBM) method was utilized in the current investigation, which can fairly and comprehensively evaluate the anatomical differences in gray matter (GM) throughout the entire brain (Ashburner and Friston, 2000). VBM is considered to be a better method than others to compare the relative concentration of GM (that is, the proportion of GM to other tissue types in the region) (Ashburner and Friston, 2000) and to detect brain abnormalities more sensitively at the voxel level (Khan et al., 2015; Goto et al., 2022). A prospective study (before versus after pregnancy) showed that pregnancy is related to significant changes in the human brain structure, and primiparous women have been found to experience a symmetrical pattern of extensive gray matter volume (GMV) reduction throughout pregnancy, particularly in regions subserving social cognition, such as specific regions of the bilateral lateral prefrontal and temporal cortex (Hoekzema et al., 2017). Previous studies have found that older individuals with hypertension is linked to lower regional GMV, which mainly affects the frontal and medial temporal lobes, including the amygdala and hippocampus (Schaare et al., 2019). In addition, a case control study found that women with a history of preeclampsia had lower temporal cortical GMV than women with normotensive pregnancies (Siepmann et al., 2017). These studies have shown that pregnancy, chronic hypertension and preeclampsia may all affect the GMV of the human brain. However, few studies have investigated GMV alterations in CHTN-PE patients.

With the increase in sex hormones during pregnancy, women often show cognitive and subjective emotional changes, as well as a slight decline in cognitive function (Rehbein et al., 2022). Studies have found that women experienced local GMV reduction during pregnancy, and lower GMV is independently related to poor cognitive and locomotive performance (Hoekzema et al., 2017; Beauchet et al., 2019; Rehbein et al., 2022). Therefore, decreased GMV leads to cognitive dysfunction, which may explain why women experience emotional and cognitive changes after pregnancy. The Stroop color-word test (SCWT) is one of the most commonly used measures to evaluate a variety of cognitive functions, including the ability to inhibit cognitive interference, as well as attention, cognitive flexibility, information processing speed, working memory and visual search (Scarpina and Tagini, 2017). At present, few studies have examined the connection between GMV alterations and cognitive function in CHTN-PE patients.

The objectives of this study were, first, to discuss the characteristics of decreased GMV in pregnant healthy women and CHTN-PE patients and, second, to summarize the characterization of GMV reductions associated with cognitive dysfunction.

## 2. Materials and methods

### 2.1. Subjects

This prospective and cross-sectional study was approved by Jinan Maternity and Child Care Hospital’s institutional review board. Twenty-five CHTN-PE patients (age 32.24 ± 5.00 years), thirty-five non-pregnant healthy controls (NPHC) (age 32.29 ± 4.99 years) and thirty-five pregnant healthy controls (PHC) (age 30.34 ± 5.73 years) from July 2020 to April 2021 were enrolled in this cross-sectional study. All participants were clear about the detailed experimental procedures and signed informed consent forms. CHTN-PE patients were recruited from the obstetric ward and had to be clinically stable to complete the MRI examination. No subjects were diagnosed with eclampsia, either before or after participating in the study. All pregnant participants had blood pressure (BP) measured, blood and urine sampling, and MRI examinations within 12 h after study enrollment. BP was measured in the right arm after subjects had been supine for 15 min. Routine blood and urinary tests were performed in all pregnant participants. The CHTN-PE patients included in this study did not demonstrate hypertension crisis after the evaluation of clinicians and could be examined by MRI. MRI examinations and cognitive assessments for all pregnant women were completed after admission, before delivery, and under clinically stable conditions.

**FIGURE 1:**
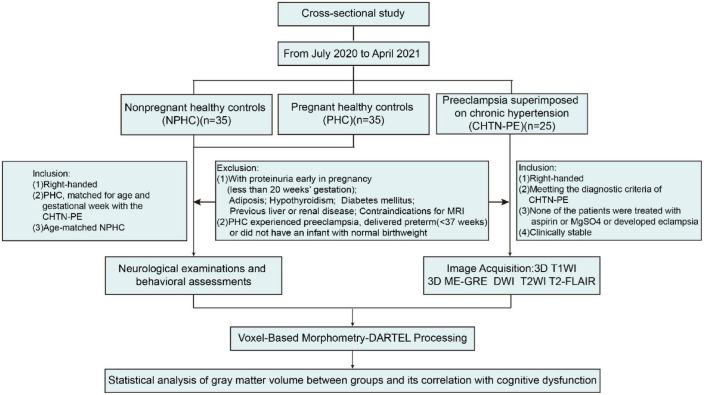
Steps for subject selection or study participation and inclusion and exclusion criteria.

The inclusion and exclusion criteria are shown in Figure 1. The inclusion criteria of CHTN-PE were as follows: (1) All participants were right-handed, and all CHTN-PE patients met the diagnostic criteria of CHTN-PE: chronic hypertensive pregnant women with new onset proteinuria (0.3 g of protein or more in a 24 h specimen) after 20 weeks gestation and without proteinuria early in pregnancy (less than 20 weeks gestation); had proteinuria before pregnancy with a significant increase in proteinuria after pregnancy or a further increase in blood pressure; or had serious manifestations, such as liver and kidney damage, pulmonary edema, epigastric pain, headache, nervous system abnormalities or visual impairment (Wilkerson and Ogunbodede, 2019; Kametas et al., 2022). (2) None of the patients were given aspirin or MgSO4 or developed eclampsia, either before or after participation in the study. (3) All patients were clinically stable for transport to the MRI facility. The following were the healthy controls’ inclusion criteria: (1) PHC, matched for gestational week and age with the CHTN-PE group, were recruited through informational posters at antenatal outpatient clinics in the Jinan Maternal and Child Care Hospital; (2) NPHC, matched for age, were recruited from the local community. The general exclusion criteria for all subjects were proteinuria during early pregnancy (less than 20 weeks gestation), adiposis, hypothyroidism, previous liver or renal disease, diabetes mellitus, or contraindications for MRI. This analysis eliminated PHC subjects who experienced preeclampsia, delivered an infant prematurely (before 37 weeks), or did not have an infant with normal birthweight (between two standard deviations of the mean birthweight for gestational age and sex).

### 2.2. Cognitive assessment

All participants underwent detailed neurological examinations by professional neurologists. The Beijing version of the Montreal Cognitive Assessment (MoCA) was used to evaluate cognitive functions (Tian et al., 2020), which is a one-page 30-point test administered in 10 min, including 11 items and it is an assessment tool for rapid screening for mild cognitive impairment (Bergeron et al., 2017). The optimal cut-off for detecting cognitive impairment points was 13/14 for illiterate individuals, 19/20 for individuals with 1–6 years of education, and 24/25 for individuals with 7 or more years of education (Lu et al., 2011). One point is added if the education years is less than 12. The SCWT is one of the most commonly used measures to evaluate a variety of cognitive functions and consists of three parts: Stroop word, Stroop color, and Stroop color-word. The subjects are required to read the names of colors printed in black ink in the first part, and they are also asked to name various color patches in the second part. Conversely, in the last part, each color-word is printed in an inconsistent color ink, and subjects are asked to quickly identify the color of the ink instead of reading the words (Scarpina and Tagini, 2017; Ktaiche et al., 2022). For the SCWT, higher scores were associated with more severe symptoms (Radetz et al., 2021). The implementers of all the above tests had received qualified professional training. The examination was conducted after scanning, and the total examination time shall not exceed 15 min.

### 2.3. Image acquisition

Magnetic resonance imaging scanning was performed on a 1.5-T MR scanner (Achieva, Philips Healthcare, Best, Netherlands) using a 16-channel head coil for signal reception. A three-dimensional (3D) T1-weighted (T1W) high-resolution sequence with the following parameters was used to generate the structural images [repetition time (TR): 25 ms; echo time (TE): 6.1 ms; inversion time (TI): 900 ms; flip angle: 30^°^; and isotropic voxel size: 1 mm^3^]. Furthermore, T2-weighted (T2W) turbo spin echo, T2W fluid-attenuated inversion recovery (FLAIR) and diffusion-weighted images (DWI) were obtained and used to identify brain abnormalities. The average scanning time of each sequence is about 5 min, and the total imaging time did not exceed 20 min.

### 2.4. Voxel-based morphometry (VBM)-DARTEL processing

**FIGURE 2:**
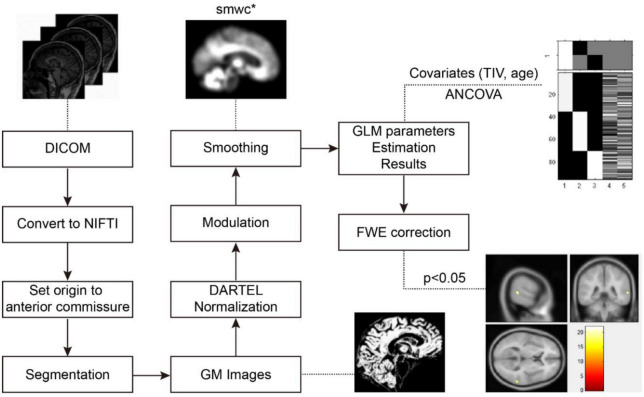
The processing pipeline of voxel-based morphometry (VBM)-DARTEL analysis using SPM. DARTEL, diffeomorphic anatomical registration through exponentiated lie algebra; FWE, familywise error; ANCOVA, one-way analysis of covariance; GM, gray matter; SPM, statistical parametric mapping.

Three-dimensional T1W images were processed using VBM with diffeomorphic anatomical registration through exponentiated lie algebra (DARTEL) (Ashburner, 2007) based on the statistical parametric mapping (SPM8)^1^ toolbox after data acquisition (pipeline shown in Figure 2). Using a unified tissue-segmentation procedure, the images were segmented into GM, white matter (WM), and cerebrospinal fluid (CSF) after image-intensity non-uniformity correction. The study-specific GM templates were created by the DARTEL algorithm, and all GM images were warped to the template and then normalized to Montreal Neurological Institute (MNI) standard space. Then, the normalized GM images were modulated to correct volume changes, and the generated gray matter volume (GMV) images were smoothed with the isotropic Gaussian kernel of 8 mm full-width at half-maximum.

### 2.5. Statistical analysis

A two-sample *t*-test was applied to assess differences in gestational weeks between the NPHC and PHC groups, and one-way analysis of variance (ANOVA) and least significant difference (LSD) *post-hoc* tests were used to examine differences in other demographic and clinical parameters among the three groups using the Statistical Package for the Social Sciences (SPSS Inc, v24.0, Chicago, IL, USA). The SPM8 toolbox was used to perform one-way analysis of covariance (ANCOVA) with age and total intracranial volume (TIV) as covariates to compare GMV differences among the three groups, and the family-wise error (FWE) correction (voxel-wise *p* < 0.05, cluster size > 50) based on Random Field Theory (RFT) method was applied to control multiple comparisons (Kilner et al., 2005). Once significant GMV differences among the three groups were identified in any brain clusters, the mean GMV values within the clusters were extracted. Then, the mean GMV value of each significant cluster was analyzed using ANCOVA and LSD post-hoc analyses. SPSS was used to calculate Pearson’s correlations between the mean GMV and cognitive parameters in all subjects, and false discovery rate (FDR) corrected *p*-value < 0.05 was set as the significance threshold.

To verify that GMV can be used as a diagnostic neuroimaging biomarker to distinguish CHTN-PE patients from the PHC and NPHC groups, the mean GMV value of each significant cluster was also used for receiver operating characteristic (ROC) curve analysis using MedCalc Statistical Software.^2^ According to the ROC curve, the optimal cut-off point was found based on the Youden index (Hajian-Tilaki, 2013, 2018). Then, sensitivity and specificity were calculated based on the cut-off points.

## 3. Results

### 3.1. Demographic and clinical characteristics

**FIGURE 3:**
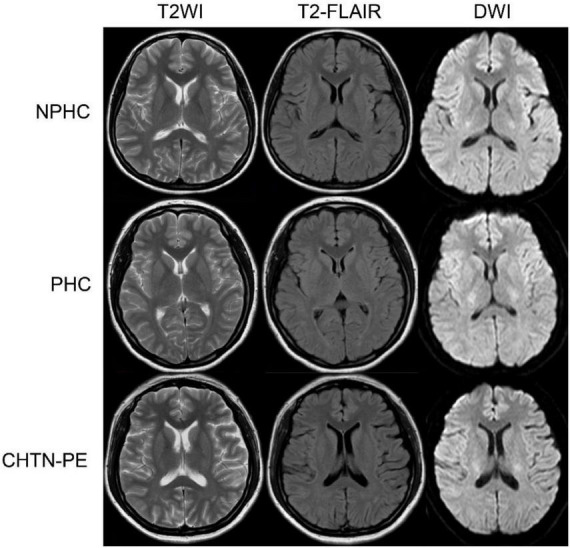
Non-pregnant healthy controls, (NPHC) a 35 years old female. Pregnant healthy controls (PHC), a 28 years old female who was pregnant for the first time for 28 weeks. Preeclampsia superimposed on chronic hypertension (CHTN-PE), a 33 years old female who was pregnant for the first time for 38 weeks. No abnormality is found in the routine magnetic resonance imaging in the three groups. T2WI, T2-weighted imaging; T2-FLAIR, T2-fluid-attenuated inversion recovery; DWI, diffusion-weighted imaging.

The clinical characteristics of the study participants are summarized in Table 1. Differences in age, body mass index, blood pressure, WM volume, CSF volume, total brain volume, MoCA and SCWT scores were evaluated using ANOVA with LSD *post-hoc* tests, and a two-sample *t*-test was performed to assess differences in gestational week. The mean MoCA and Stroop word scores were significantly different among the three groups. The CHTN-PE group had lower MoCA scores and higher Stroop word scores than the NPHC and PHC groups, and the PHC group had lower MoCA scores and higher Stroop word scores than the NPHC group. No significant differences were found in age, gestational week, Stroop color and Stroop color-word scores among the three groups. No abnormality was found in routine magnetic resonance imaging in three groups (Figure 3).

**TABLE 1:** Clinical characteristics of the participants.

Variables	NPHC *n* = 35	PHC *n* = 35	CHTN-PE *n* = 25	F/t value	*P*-value	*P*-value (*post-hoc*)		
						**NPHC vs.** **CHTN-PE**	**PHC vs.** **CHTN-PE**	**NPHC vs. PHC**
Female (n)	35	35	25	–	–	–	–	–
Age (years)	32.29 ± 4.99	30.20 ± 5.38	32.24 ± 5.00	1.787	0.173^a^	–	–	–
Gestational week (week)	–	34.24 ± 4.10	34.92 ± 4.23	0.884	0.380^t^	–	–	–
Body mass index (kg/m^2^)	22.00 ± 3.02	27.16 ± 4.44	31.67 ± 4.13	46.045	<0.001^a^	<0.001	<0.001	<0.001
Weight (kg)	57.74 ± 7.41	71.36 ± 11.83	81.94 ± 12.85	38.303	<0.001^a^	<0.001	<0.001	<0.001
Systolic pressure (mmHg)	111.60 ± 11.60	113.57 ± 11.20	162.00 ± 15.07	145.001	<0.001^a^	<0.001	<0.001	0.510
Diastolic pressure (mmHg)	67.97 ± 9.05	73.68 ± 7.96	104.48 ± 9.67	137.139	<0.001^a^	<0.001	<0.001	0.008
Mean atrial pressure (mmHg)	82.51 ± 9.04	86.98 ± 8.13	123.66 ± 10.68	167.303	<0.001^a^	<0.001	<0.001	0.045
WMV	546.82 ± 45.96	542.52 ± 44.85	552.65 ± 47.76	0.353	0.703^a^	0.629	0.697	0.697
CSF volume	172.21 ± 17.19	187.07 ± 19.90	181.59 ± 22.20	5.121	0.008^a^	0.071	0.289	0.002
Total brain volume	1277.60 ± 85.62	1269.31 ± 75.07	1288.04 ± 81.56	0.392	0.677^a^	0.623	0.378	0.669
MoCA	29.74 ± 0.66	29.11 ± 0.90	28.08 ± 1.22	24.153	<0.001^a^	<0.001	<0.001	0.004
Stroop word	18.31 ± 3.62	21.23 ± 4.05	23.56 ± 3.50	14.661	<0.001^a^	<0.001	0.020	0.002
Stroop color	29.17 ± 6.28	32.26 ± 6.83	32.88 ± 6.47	2.963	0.057^a^	–	–	–
Stroop color-word	56.91 ± 11.32	63.03 ± 11.83	59.16 ± 12.51	2.383	0.098^a^	–	–	–

Data are displayed as the mean ± standard deviation.

^a^Analysis of variance (ANOVA) test.

^t^Two-sample *t*-test; NPHC, non-pregnant healthy control; PHC, pregnant healthy control; CHTN-PE, preeclampsia superimposed on chronic hypertension; kg, kilograms; MoCA, Montreal Cognitive Assessment; WMV, white matter volume; CSF, cerebrospinal fluid.

### 3.2. GMV differences among groups

**FIGURE 4:**
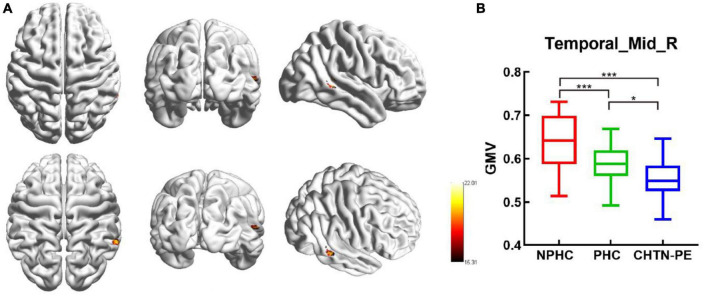
Significantly altered gray matter volume (GMV) among groups. **(A)** Clusters with significantly altered GMV among groups [family-wise error (FEW) corrected, *p* < 0.05]. **(B)** Mean GMV values in the cluster of the right middle temporal gyrus among NPHC, PHC, and CHTN-PE patients. CHTN-PE, preeclampsia superimposed on chronic hypertension; NPHC, non-pregnant healthy controls; PHC, pregnant healthy controls; Temporal_Mid_R, right middle temporal gyrus. *P < 0.05; ***P < 0.001.

Using ANCOVA, we found significant GMV differences in a cluster of the right MTG among the three groups (FWE corrected, *p* < 0.05). The mean GMV values within the right MTG cluster were extracted, and follow-up LSD *post-hoc* tests revealed that the CHTN-PE group had significantly lower GMV than the NPHC and PHC groups and that the PHC group had lower GMV than the NPHC group in the right MTG cluster (shown in Table 2 and Figure 4).

**TABLE 2:** Significantly altered gray matter volume (GMV) among the non-pregnant healthy control (NPHC), pregnant healthy control (PHC), and preeclampsia superimposed on chronic hypertension (CHTN-PE) groups.

Brain regions	Peak MNI (X, Y, Z)		Cluster size	F	Z	*P*		Volume (ml): mean ± SD		
								**NPHC**	**PHC**	**CHTN-PE**
Temporal_Mid_R	61	−42	6	97	15.30	22.01	<0.001	0.64 ± 0.06	0.58 ± 0.04	0.55 ± 0.05

CHTN-PE, preeclampsia superimposed on chronic hypertension; NPHC, non-pregnant healthy controls; PHC, pregnant healthy controls; Temporal_Mid_R, right middle temporal gyrus. Cluster size, the number of voxels in the (identified significant) cluster. The familywise error (FWE) method was used for correction.

### 3.3. Diagnostic accuracy and performance of GMV

**FIGURE 5:**
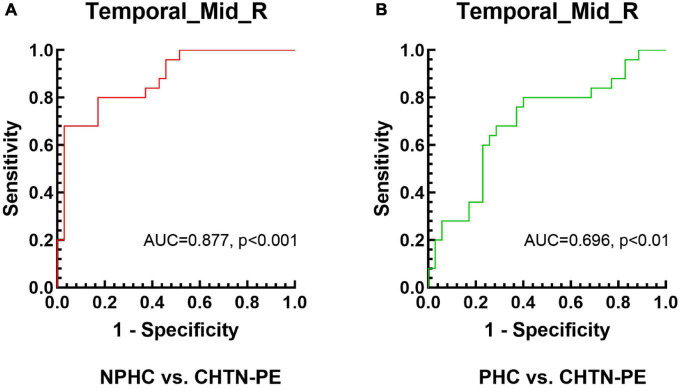
Receiver operating characteristic (ROC) curve analysis results. ROC curves for the mean gray matter volume (GMV) values of the right middle temporal gyrus that significantly distinguish the CHTN-PE group from the **(A)** NPHC and **(B)** PHC groups. CHTN-PE, preeclampsia superimposed on chronic hypertension; NPHC, non-pregnant healthy controls; PHC, pregnant healthy controls; Temporal_Mid_R, right middle temporal gyrus.

From the ROC curves, the GMV values of the right MTG cluster can distinguish the CHTN-PE group from the NPHC and PHC groups; all AUC values had a significance level of *p* < 0.01. According to the Youden index, the cut-off values for each value in the various ROIs were chosen, and the specifics are shown in Table 3, Figure 5.

**TABLE 3:** The statistics of receiver operating characteristic (ROC) curve analysis for the cluster of right middle temporal gyrus (MTG) that distinguishes preeclampsia superimposed on chronic hypertension (CHTN-PE) patients from the non-pregnant healthy controls (NPHC) and pregnant healthy controls (PHC) groups.

	AUC (95% CI)	*P*-value	Cut-off value	Sensitivity	Specificity
NPHC vs. CHTN-PE	0.877 (0.766–0.947)	<0.001	0.564	0.680	0.971
PHC vs. CHTN-PE	0.696 (0.564–0.808)	<0.01	0.584	0.800	0.600

CHTN-PE, preeclampsia superimposed on chronic hypertension; NPHC, non-pregnant healthy controls; PHC, pregnant healthy controls; AUC, area under the curve; CI, confidence interval.

### 3.4. Correlations between neuroimaging and cognitive function

**FIGURE 6:**
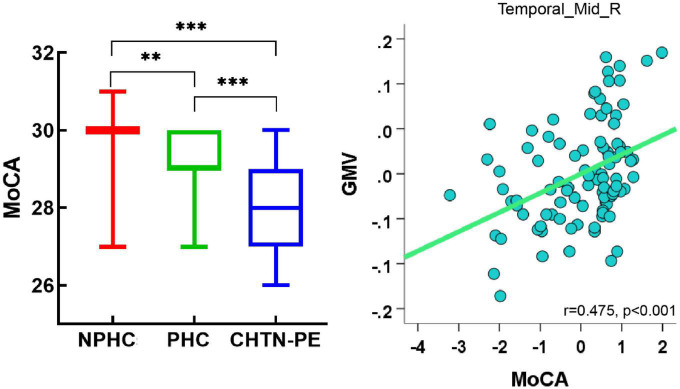
Correlations between the mean GMV of the right middle temporal gyrus and Montreal Cognitive Assessment (MoCA) scores in all subjects. Of note, the coordinate values of both the X axis (MoCA scores) and Y axis (GMV) do not reflect the initial values of these variables when considering age and total intracranial volume (TIV) as covariates. Significance was set to *p* < 0.05, false discovery rate (FDR) corrected for multiple comparison. GMV, gray matter volume; Temporal_Mid_R, right middle temporal gyrus; CHTN-PE, preeclampsia superimposed on chronic hypertension; NPHC, non-pregnant healthy controls; PHC, pregnant healthy controls. ^**^p < 0.01, ^***^p < 0.001.

**FIGURE 7:**
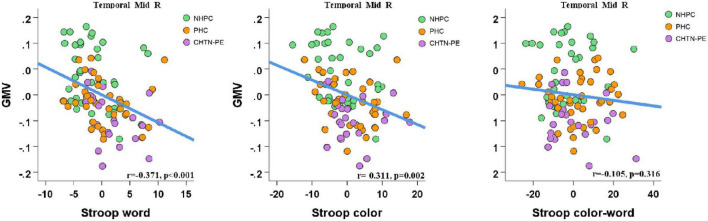
Correlations between the mean GMV of the right middle temporal gyrus and Stroop color and word test (SCWT) scores in all subjects. Of note, the coordinate values of both the X axis (SCWT scores) and Y axis (GMV) do not reflect the initial values of these variables when considering age and total intracranial volume (TIV) as covariates. Significance was set to *p* < 0.05, false discovery rate (FDR) corrected for multiple comparison. GMV, gray matter volume; Temporal_Mid_R, right middle temporal gyrus; CHTN-PE, preeclampsia superimposed on chronic hypertension; NPHC, non-pregnant healthy controls; PHC, pregnant healthy controls.

We extracted the mean GMV values within the significantly altered cluster (right MTG) for all subjects. Correlation analysis revealed a significant positive correlation between the mean GMV of the right MTG and MoCA (*r* = 0.475, *p* < 0.001; shown in Figure 6), and a significant negative correlation between the mean GMV of the right MTG and Stroop word and Stroop color scores in all subjects (separately: *r* = −0.371, *p* < 0.001; *r* = −0.311, *p* = 0.002; FDR corrected). There were no significant correlations between the GMV and Stroop color-word scores in all subjects (*r* = −0.105, *p* = 0.316) (shown in Figure 7).

## 4. Discussion

The current study applied the VBM approach to explore brain morphological alterations in pregnant women with or without CHTN-PE. In addition to CHTN-PE patients, individuals were recruited to both the PHC and NPHC groups, which is an advantage of our study, as their data can be used to provide a more systematic explanation for the alterations in brain structure of CHTN-PE patients. We found that compared with the NPHC, the PHC and CHTN-PE patients showed significantly decreased GMV in the right MTG cluster, and GMV decreased more significantly in the CHTN-PE patients. We also estimated the correlation between the regions where GMV significantly decreased and cognitive function and found that GMV in the cluster of right MTG was significantly negatively correlated with Stroop word and Stroop color scores and positively correlated with MoCA, suggesting a decline in the ability to inhibit cognitive interference, attention, information processing speed, cognitive flexibility and visual search in CHTN-PE patients. To the best of our knowledge, these data provide the first insight into the profound impact of CHTN-PE on cerebral GMV.

It has been confirmed that pregnancy results in long-lasting alterations of the human brain structure (Hoekzema et al., 2017). However, the effect of CHTN-PE on brain structure is not clear. Previous animal studies have demonstrated that modulation of the renin–angiotensin system (RAS) is the key mechanism in the development of CHTN-PE (Genest et al., 2013), and because renin is species specific for angiotensinogen cleavage, the overexpression of RAS in the uterus and placenta and the release of placental human renin into the circulation trigger preeclampsia-like symptoms in some rodents (Falcao et al., 2009). A previous study showed that cardiovascular disease-related neuronal loss was a similar burden in all women who experienced preeclampsia, which was manifested as GM atrophy (Raman et al., 2017). The occurrence of brain atrophy in chronic hypertension patients is a long-term and aging process, and young hypertension patients may not necessarily have GMV changes (Strassburger et al., 1997). Previous studies have shown that young chronic hypertension patients (<40 years) are associated with a decrease in GMV in the frontoparietal lobe, and no change in GMV in the temporal lobe has been found (Schaare et al., 2019). The subjects included in this study were young pregnant women (<40 years), therefore, we believe that the likelihood of changes in GMV of the MTG before pregnancy is minimal. We consider that CHTN-PE patients will experience neuronal loss and GM atrophy during pregnancy, resulting in the reduction of GMV in the right MTG. Besides, it was found that there was hemispheric asymmetries of GMV in left and right-handed people (Saenger et al., 2012). In order to eliminate the difference in GMV between left and right-handed people, all the subjects we included are right-handed. Rightward functional asymmetries were observed in the middle frontal and middle/superior temporal gyrus in the right-handed group (Saenger et al., 2012), so the decrease of GMV in CHTN-PE patients may first occur in the right middle frontal gyrus, superior temporal gyrus and middle temporal gyrus. Therefore, our findings that GMV in the cluster of right MTG is significantly decreased in CHTN-PE patients are also reasonable.

It has been found that laboratory indicators such as uric acid, the renin-angiotensin aldosterone system, angiogenic factors and proinflammatory markers of endothelial dysfunction have changed in women with chronic hypertension, but none of these biomarkers have been proved to be useful in the screening and diagnosis of superimposed preeclampsia (Kametas et al., 2022). By using imaging indicators, the early brain structural changes of superimposed preeclampsia can be found sensitively (Raman et al., 2017), and early identification of CHTN-PE patients from pregnant women would contribute to more intensive surveillance, more aggressive care, and hopefully better maternal and fetal outcomes (August et al., 2004). Therefore, it is also valuable to use imaging indicators to assist in the diagnosis of CHTN-PE. Analysis of the ROC curve in this study revealed that mean GMV values in the right MTG cluster had excellent classification performance for distinguishing CHTN-PE patients from healthy controls, which could be used as a potential diagnostic biomarker. The ROC curve is also used to determine the optimal cut-off value for diagnosing a disease (Nahm, 2022). According to the results of this study and combined with clinical manifestations and laboratory indicators, when GMV in the right MTG is less than 0.584, we should suspect that pregnant women may be at risk of CHTN-PE.

The MTG affects a variety of functions, including language, emotion, memory and social cognition (Xu et al., 2019), and is considered to play an important role in language-related tasks, such as vocabulary comprehension and semantic cognition (Briggs et al., 2021). Therefore, the right MTG is closely related to cognitive and executive functions and it is reasonable for CHTN-PE patients with significantly reduced GMV in the right MTG to have lower MoCA scores and higher SCWT scores. MoCA is more commonly used in the field of dementia and in the diagnosis of mild cognitive impairment, with better sensitivity in this population (Bergeron et al., 2017). SCWT can efficiently evaluate a variety of cognitive functions, including the ability to inhibit cognitive interference, as well as cognitive flexibility, attention, information processing speed, working memory, and visual search (Scarpina and Tagini, 2017). The completion time of SCWT is only 2 or 3 min, fully considering the tolerance of CHTN-PE patients. Therefore, we believe that the SCWT could better reflect the cognitive dysfunction caused by decreased GMV in the right MTG.

Stroop color and word test scores may be affected by the impairment of speech motor function and the decline in cognitive flexibility (Ktaiche et al., 2022). Multiple regression analysis of Stroop word and Stroop color data suggested that Stroop word scores were predicted by the speed of visual search and Stroop color scores were predicted by the speed of visual search and working memory (Periáñez et al., 2021), both of which were related to the speed of visual search, and visual search is closely related to language comprehension (Huettig et al., 2011; Chiu and Spivey, 2014). According to our study, GMV decreases in the right MTG cluster in the PHC and CHTN-PE groups may lead to declines in language and visual comprehension, which could increase Stroop color and Stroop word scores. Hence, it is reasonable that GMV in the right MTG cluster has a significant negative correlation with Stroop word and Stroop color scores. These findings may explain the decline in speech motor function and cognitive flexibility after pregnancy. Moreover, the additional complication of CHTN-PE in pregnant women might have caused a more obvious GMV decrease and might have resulted in more severe related cognitive impairment; this was an important finding of our study.

In the Stroop color-word task, participants were required to perform a task that was less automated (naming ink color) while suppressing interference from a task that was more automated (reading words) (Scarpina and Tagini, 2017). In fact, a previous study found that in the incongruous condition, the subjects also experienced non-pathological slowing down of reading speed even if they understood the task correctly (Scarpina and Tagini, 2017). In other words, the healthy controls may also have non-pathological slow reading speed because of the incongruous condition, resulting in high scores. Although the psychological structures associated with Stroop color-word scores include cognitive control, speech and semantic fluency, interference control, cognitive flexibility or working memory, some studies have found that after controlling visual search and perceptual speed factors, the association between Stroop color-word task and cognitive flexibility disappeared (Periáñez et al., 2021). Besides, the association between Stroop color-word task and verbal fluency may be more related to shared executive control, rather than language skills (Aita et al., 2019; Periáñez et al., 2021). These findings may provide an explanation for our results that there was no difference in Stroop color-word scores among the three groups and no correlation with GMV in the right MTG.

Although VBM is a typical method for measuring GMV, a recent study has shown that the combination of VBM and surface-based morphometry can complement the detection of cortical morphological changes and detect cortical morphological changes more accurately (Goto et al., 2022). Additionally, a brand-new method known as multivariate pattern analysis has been proven to be more sensitive and accurate in capturing the cerebral cortex’s function (Haxby, 2012). Therefore, more detailed brain structure and function analysis of CHTN-PE patients can be carried out by combining more advanced image segmentation methods and artificial intelligence in the future. Second, the study’s sample size was small, and only GMV alterations in CHTN-PE patients were studied. Whether there are abnormalities in cerebral blood flow (CBF) and cerebral oxygen metabolic rate (CMRO_2_) in CHTN-PE patients has not been evaluated. CBF and CMRO_2_ can be obtained by adding multi-delay arterial spin labeled images (Zhang et al., 2020). Therefore, more imaging methods and larger sample sizes are needed in future studies. We are further expanding the sample size collection and planning to conduct further research on chronic hypertension without superimposed preeclampsia, and conduct longitudinal studies of pregnant women with chronic hypertension.

## 5. Conclusion

In conclusion, we explored the possible pathogenesis of CHTN-PE by analyzing GMV alterations in CHTN-PE patients. The results showed that GMV in a cognitive-related cerebral regions, the right MTG, was significantly decreased in CHTN-PE patients, and combined with the SCWT scores, it could provide an explanation for related cognitive dysfunction in CHTN-PE patients. These findings might help us better understand the relationship between brain GMV alterations and the pathophysiology of CHTN-PE.

## Data availability statement

The raw data supporting the conclusions of this article will be made available by the authors, without undue reservation.

## Ethics statement

The studies involving human participants were reviewed and approved by the Ethical Committee of the Institutional Review Board (IRB) of Jinan Maternity and Child Care Hospital (20190618). The patients/participants provided their written informed consent to participate in this study.

## Author contributions

CS wrote the main manuscript text. CS and HW performed the statistical analysis. JH, TC, YG, and YW prepared the clinical data and imaging data. LY and LG revised the main manuscript text. All authors reviewed the manuscript.

## Footnotes

1
http://www.fil.ion.ucl.ac.uk/spm

2
https://www.medcalc.org
